# Disulfiram: Mechanisms, Applications, and Challenges

**DOI:** 10.3390/antibiotics12030524

**Published:** 2023-03-06

**Authors:** Jenna Lanz, Nicholas Biniaz-Harris, Mara Kuvaldina, Samta Jain, Kim Lewis, Brian A. Fallon

**Affiliations:** 1 Lyme & Tick-Borne Diseases Research Center, Department of Psychiatry, Vagelos College of Physicians and Surgeons, Columbia University Irving Medical Center, Columbia University, New York, NY 10032, USA; 2 Antimicrobial Discovery Center, Department of Biology, Northeastern University, Boston, MA 02115, USA; 3 New York State Psychiatric Institute, New York, NY 10032, USA

**Keywords:** disulfiram, Antabuse, drug repurposing, Lyme disease, risks, addiction

## Abstract

**Background**: Since disulfiram’s discovery in the 1940s and its FDA approval for alcohol use disorder, other indications have been investigated. This review describes potential clinical applications, associated risks, and challenges. **Methods**: For this narrative review, a PubMed search was conducted for articles addressing in vivo studies of disulfiram with an emphasis on drug repurposing for the treatment of human diseases. The key search terms were “disulfiram” and “Antabuse”. Animal studies and in vitro studies highlighting important mechanisms and safety issues were also included. **Results**: In total, 196 sources addressing our research focus spanning 1948–2022 were selected for inclusion. In addition to alcohol use disorder, emerging data support a potential role for disulfiram in the treatment of other addictions (e.g., cocaine), infections (e.g., bacteria such as Staphylococcus aureus and Borrelia burgdorferi, viruses, parasites), inflammatory conditions, neurological diseases, and cancers. The side effects range from minor to life-threatening, with lower doses conveying less risk. Caution in human use is needed due to the considerable inter-subject variability in disulfiram pharmacokinetics. **Conclusions**: While disulfiram has promise as a “repurposed” agent in human disease, its risk profile is of concern. Animal studies and well-controlled clinical trials are needed to assess its safety and efficacy for non-alcohol-related indications.

## 1. Introduction

Disulfiram was first synthesized in 1881 by the German chemist M. Grodzki, although at the time, his publication in *Berichte* noting the discovery received little recognition [1,2]. Fifty years later, the compound was rediscovered as an agent used to accelerate the vulcanization of rubber [2,3]. In 1937, American physician E.E. Williams noted that the workers in a rubber plant experienced negative physiological effects following the ingestion of alcohol, but it would be another decade before this unique effect of disulfiram was first investigated for clinical use [1,2,3].

In the 1940s, Danish researchers Dr. Erik Jacobsen and Dr. Jens Hald became interested in disulfiram following promising studies from British and Swedish scientists demonstrating disulfiram’s ability to cure scabies in domestic animals [1,2]. Jacobsen and Hald discovered that disulfiram chelates copper, thereby depleting the scabies parasite of its primary oxygen transporter [1,2]. They posited that this mechanism could make disulfiram an effective treatment for intestinal worms in both animals and humans [1,2]. In order to test the safety of disulfiram for clinical use, Jacobsen and Hald first tested disulfiram on themselves [1,2]. Following the experience, Jacobsen wrote that “the disulfiram tablets really changed the effect of alcohol in a most unpleasant direction [1].” In 1948, Jacobsen and Hald published an article investigating the physiological effects of disulfiram following alcohol ingestion [4].

In the original study, the participants were given 1–1.5 g of disulfiram (2–3× the current FDA-approved maximum dose of 500 mg) followed by 30–60 mL of gin and were monitored for adverse reactions [4]. Jacobsen and Hald noted the development of facial vasodilation, tachycardia, and increased pulmonary ventilation [4]. After a collaborator noted the strong smell of acetaldehyde in the lab, Hald and Jacobsen hypothesized that elevated acetaldehyde levels may be responsible for disulfiram’s negative physiologic effects when combined with alcohol [1,2,4]. They tested this hypothesis by performing continuous intravenous infusions of acetaldehyde into participants until the blood acetaldehyde levels reached those found in disulfiram users after alcohol intake. At these high levels, the participants experienced the same negative effects seen in disulfiram [4]. From these experiments, they concluded that disulfiram likely worked by blocking the normal elimination of alcohol.

From 1948–1953, there were nearly 150 publications from Europe and the United States studying disulfiram, its effects, and its potential utility as a treatment for alcohol use disorder [1]. In 1951, disulfiram became the first FDA-approved treatment for alcohol use disorder in the United States [3]. At the time of its approval, common treatment dosages of disulfiram ranged from 1000–3000 mg daily [2,3]. At these high doses, the drug became notorious for causing serious side effects as well as case reports of death resulting from severe disulfiram–ethanol reactions [2,3]. In the late 1960s, the use of subcutaneous implants of disulfiram became popular as a method for ensuring compliance in alcohol use disorder treatment [3,5]. However, due to insufficient evidence regarding the utility and safety of this method of drug delivery, disulfiram implants have never been licensed for use in the United States [3,5].

Since the introduction of other FDA-approved pharmacological treatments such as naltrexone and acamprosate, disulfiram has fallen out of favor as a treatment for alcohol use disorder [6]. However, it still remains a drug of interest in many facets of clinical medicine, as new mechanisms and possible uses continue to be discovered. This narrative review describes the many promising potential applications for the clinical use of disulfiram as well as the many challenges related to its pharmacokinetic variability, safety, and potential adverse events.

## 2. Results

In total, 196 sources in English were included in this review, covering a 74-year time span (1948–2022). The treatment studies and clinical series on the use of disulfiram in humans are summarized in Supplementary Table S1.

### 2.1. Mechanism(s) of Action 

Disulfiram, or tetraethylthiuram disulfide, is an electrophilic quaternary ammonium compound that has been shown to potently inhibit a diverse collection of metabolic enzymes [7,8,9,10,11]. Several important mechanisms of inhibition have been identified, although ongoing studies continue to describe new possibilities [10,12,13].

**Figure 1:**
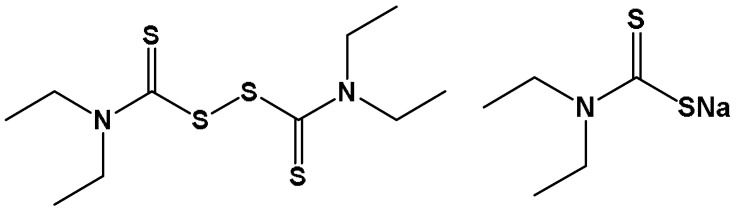
Chemical Structure of Disulfiram (**left**) and Sodium DDTC (**right**). “Structure of disulfiram” by NEUROtiker is in the public domain and was accessed via Wikimedia Commons.

In the 1940s, Jacobsen and Hald first described disulfiram’s ability to chelate metal ions [1,2]. Later studies identified diethyldithiocarbamate (DDTC) as the particular disulfiram metabolite responsible for the drug’s chelating abilities [14,15]. DDTC is the reduced form of disulfiram (Figure 1) and a powerful chelator of transition divalent metal ions, including copper and zinc [10,16]. Through the chelation of essential cofactors, DDTC can impair the function of metal-containing enzymes, including aldehyde dehydrogenase (ADH), carboxylesterase, cholinesterase, superoxide dismutase, and dopamine β-hydroxylase (DBH) [17,18]. The complex of copper and diethyldithiocarbamate Cu (DTC)_2_ is considered the active metabolite responsible for the anticancer properties of disulfiram (see Section 2.6).

Disulfiram contains a disulfide bond that is readily reduced by the free sulfhydryl groups of proteins via the thiol–disulfide exchange reaction. This irreversible reaction covalently modifies cysteine residues on target enzymes, causing the inhibition of the enzymatic function and resulting in the release of a DDTC molecule [19,20]. Since the 1940s, several enzymes including aldehyde dehydrogenase, methyltransferase, urease, and kinase have been proven susceptible to disulfiram inhibition via this mechanism [11,20,21].

Disulfiram is also capable of altering the intracellular environment by directly or indirectly manipulating the concentration of reactive oxygen species [12,22]. Reactive oxygen species (ROS) are generated by cells endogenously during the process of oxidative phosphorylation or in response to foreign pathogens [23]. When the concentration of ROS overcomes the antioxidant response of the cell, the cell becomes oxidatively stressed [23]. Oxidative stress leads to damage of cellular components such as DNA, proteins, and lipid membranes, and may ultimately result in cell death [23]. The chelation reaction between DDTC and metal ions is thought to trigger the release of ROS within a cell, thereby potentially causing cellular damage or death [16,24]. Notably, some studies have also identified antioxidant properties of disulfiram that are protective against the generation or dissemination of ROS, and ultimately may be useful in reducing inflammation in certain disease states [7,12,25]. Specifically, it has been reported that thiol compounds such as disulfiram are capable of scavenging oxygen radicals, thereby depleting them from the intracellular environment [7,25]. Additional research is needed to clarify the pro- and antioxidant properties of disulfiram and its metabolites.

### 2.2. Drug Characteristics and Pharmacokinetics

Disulfiram is an orally administered drug with standard doses ranging from 250–500 mg daily and a maximum recommended daily dose of 500 mg [3,26]. Following oral disulfiram intake, 80–90% of the dose is absorbed in the gastrointestinal tract [27,28]. Due to its high lipid solubility, disulfiram and its metabolites are widely distributed into adipose tissue throughout the body and readily cross the blood–brain barrier [8,29,30]. In a study involving the administration of radiolabeled disulfiram to rats, the drug was identified in the kidneys, pancreas, liver, GI tract, fat, blood, and brain in decreasing order, among other locations [31].

The pharmacokinetics (PK) of disulfiram are not well understood. Various studies have investigated the half-life elimination of disulfiram and its metabolites in human volunteers and animals. There is a paucity of comprehensive PK data from these studies, due in part to the limitations of detection methods and to disagreements in the field regarding disulfiram detection in samples.

Disulfiram is extensively metabolized in vivo and is rapidly biotransformed into metabolic products that are responsible for its clinical uses [32]. Once disulfiram is reduced, all further metabolism takes place via DDTC, which has three possible metabolic fates:

1. Spontaneous degradation: DDTC is acid-labile and its half-life is a linear function of its pH [33]. In an acidic environment, such as the stomach, DDTC is decomposed to diethylamine and carbon disulfide (CS_2_). The CS_2_ can be detected in the blood and breath of humans after the administration of disulfiram [34,35]. After the oral administration of 250 mg of disulfiram, the half-life of CS_2_ is roughly 12 h [35]. CS_2_ has been identified in the breath 72 h after administration [36].
2. Formation of glucuronide: The formation of glucuronide is brought about by the enzyme glucuronosyltransferase, which is highly expressed in the liver. The glucuronide of DDTC accounts for 2–11% of the administered disulfiram dose that is excreted in the urine [36,37].
3. Formation of methyl esters: The enzymes thiol methyltransferase (microsomal) and possibly thiopurine methyltransferase (cytosolic), found in the liver and several other tissues including the GI tract, lungs, and kidneys, catalyze the methylation of DDTC. This methylation reaction leads to the formation of the important lipophilic metabolite DDTC-Me (methyl diethyldithiocarbamate). DDTC-Me is further oxidized to DETC-Me (methyl diethylthiocarbamate), which is then biotransformed into additional metabolites that aid in the irreversible inhibition of the ALDH enzyme [32,38,39,40,41,42,43].

Studies evaluating the elimination rates of disulfiram and some of its metabolites in humans are represented in Table 1. A rapid reduction of the initial dose of administered disulfiram or DDTC takes place in the plasma. In some cases, this rapid decline is followed by a longer terminal elimination phase [36,44,45]. The in vitro half-life of disulfiram when added to plasma is 2 to 4 min [46,47]. Rat and mouse studies have reported the half-life of disulfiram to range from 10 to 87 min, though in most studies it is below the level of detection [45,48,49]. In human studies, the half-lives of disulfiram and DDTC are estimated at 7 h and 15 h, respectively [36,50]. This wide range indicates significant inter-subject variability in the levels of disulfiram and its metabolites following oral administration. Additional studies have identified substantial and unexplained differences in plasma disulfiram levels between subjects [36,51]. One 1970s study utilizing gas chromatography found that after the administration of 250 mg of disulfiram in humans, the plasma concentration averaged 590 ± 434 ng/mL, with a range of 30–1830 ng/mL [52]. A subsequent study from 1991 using high-performance liquid chromatography found that after the administration of 200 mg of disulfiram in humans, the median plasma DDTC-Me concentration was 15.02 ng/mL with a range of 1.63–77.08 ng/mL [53]. While these two studies analyzed different metabolites, both studies indicated substantial inter-subject variability in the metabolism of disulfiram.

In a recent study, HIV+ antiretroviral therapy (ART)-suppressed participants were dosed with disulfiram and the plasma concentrations of disulfiram and four metabolites were measured using ultra-performance liquid chromatography–tandem mass spectrometry (UPLC-MS/MS). The complete elimination of disulfiram by 72 h was observed. The non-linear elimination of disulfiram was proposed with a clearance of 0.53 L/h, volume of distribution of 1.3 L, and absorption constant of 0.08/h; the half-life was not measured since disulfiram was proposed to not follow first-order elimination kinetics. Additionally, high inter-subject variability was observed, as seen in previous studies [44]. Although the reason for this variability has not been established, it may stem from differences in patient metabolism, lipid content, enterohepatic circulation, or protein-binding capacity in the plasma [36]. Moreover, linear increases in disulfiram doses have been found to supra-proportionally increase the plasma disulfiram levels; possible explanations include increased bioavailability at higher doses and the saturation of cytochrome P450 enzymes [44].

The high degree of unpredictability in plasma disulfiram levels poses a challenge in the practical application of disulfiram in the treatment of humans, as such variability would impact both its efficacy and safety profile. This variability likely accounts for the observation in one study of 63 individuals that a daily dose of 200–300 mg of disulfiram did not elicit a disulfiram–ethanol reaction in nearly half of individuals exposed to alcohol; in some, even a 500 mg dose was insufficient to elicit this reaction [54]. This variability in serum levels between patients may also account for the rare but serious risks that occur even at the lower dose of 250 mg daily. With only a few pharmacokinetic studies performed in humans, ambiguity regarding inter-subject variability impedes our understanding of disulfiram’s efficacy and safety.

### 2.3. Use in Addiction

#### 2.3.1. Disulfiram for Alcohol Use Disorder

In 1948, Jacobsen and Hald were the first to describe disulfiram’s inhibition of the enzyme acetaldehyde dehydrogenase (ALDH) [2]. In the metabolism of alcohol, ethanol is first converted to acetaldehyde by the enzyme alcohol dehydrogenase (ADH) [55]. Under normal physiologic circumstances, this toxic intermediate is then quickly converted to acetic acid by the enzyme ALDH. The inhibition of ALDH by disulfiram causes buildup of acetaldehyde, resulting in an aversive reaction that consists of flushing, headache, and nausea [3]. It is this unpleasant “hangover” effect after alcohol consumption that led clinicians to use disulfiram as an aversive therapy in patients with alcohol use disorder (AUD).

Clinical trials have reported mixed results regarding disulfiram’s efficacy in helping patients achieve abstinence from alcohol intake, although many of the early clinical studies have been criticized for their poor study design [3]. Most studies lacked a proper control group, adherence was frequently not reported or was reported as poor, and participants could not be blinded to the treatment group due to the disulfiram–ethanol effect [3,56]. The most cited disulfiram trial was conducted by the Veterans Administration in the 1980s [3]. This blinded, placebo-controlled randomized trial found no statistically significant difference in abstinence from alcohol intake between participants on 250 mg daily of disulfiram, 1 mg daily of disulfiram, or placebo [57]. However, the authors judged that the compliance rate was just 20% among the 577 men who completed the study and 23% among those in the therapeutic disulfiram group [57]. Due to these compliance issues, other trials have compared supervised disulfiram administration to unsupervised administration [56]. These studies found that patients who were instructed to receive disulfiram under the supervision of a spouse or physician had improved abstinence compared to patients who were instructed to take disulfiram unsupervised [56,58,59]. Although these studies lacked proper controls and included concomitant interventions, making the findings more difficult to interpret, reviews still endorse the use of supervised disulfiram treatment for AUD [3,60,61]. A systematic review of randomized clinical trials of pharmacotherapy for AUD found that disulfiram increased abstinence (RR = 1.77; 95% CI, 1.38–2.27) and decreased heavy drinking (RR = 0.19; 95% CI, 0.10–0.34) compared to placebo. At the same time, disulfiram caused substantially more dropouts compared to the placebo group (RR = 2.45; 95% CI, 1.02–5.88) [62]. A Bayesian network meta-analysis of treatment outcomes in RCTs of AUD and comorbid depressive symptoms found that disulfiram was the most effective medication in maintaining remission and increasing the number of abstinence days [63].

A comprehensive survey of addiction medicine specialists conducted by Ehrie et al. found that the use of disulfiram in the treatment of AUD has decreased over the past two decades [6]. The prominent reasons reported for reduced use included providers viewing disulfiram as less efficacious than other AUD medications and concerns related to its side effects [6]. Furthermore, the clinical practice guidelines recommend against the use of disulfiram in patients with alcoholic liver disease due to hepatotoxicity, which represents a major limitation for many AUD patients [64,65]. While the use of disulfiram in the treatment of AUD may be on the decline, disulfiram continues to be investigated for use in the treatment of other addiction disorders.

#### 2.3.2. Disulfiram for Cocaine Addiction

Disulfiram is also known to inhibit dopamine β-hydroxylase (DBH), the enzyme responsible for the conversion of dopamine to norepinephrine [3,66,67,68]. Blocking this enzyme causes an accumulation of dopamine within the central nervous system [8,67]. It is thought that this increase in dopamine may be used to correct the dopamine “deficit” found in patients with cocaine use disorder [26]. Ordinarily, cocaine use causes an increase in dopamine release into synaptic clefts within the central nervous system, thereby producing a euphoric response [69]. However, chronic cocaine use leads to tolerance of the dopaminergic system, resulting in a decreased dopamine responsiveness to stimuli [69]. Disulfiram-induced dopamine release in chronic cocaine users is thought to incite undesirable effects with cocaine use such as anxiety, depression, and paranoia [70]. Similar to the ethanol–disulfiram reaction, this negative reaction is thought to decrease cravings and increase abstinence from cocaine use in these patients [56]. Another study found that rather than increasing undesirable effects, disulfiram was effective in decreasing desirable effects of cocaine [71]. Patients receiving either 62.5 mg daily or 250 mg daily of disulfiram reported a significant decrease in “any high”, “cocaine high”, or “rush” when tested with a visual analog scale following IV cocaine administration compared to patients receiving placebo [71].

Regardless of the mechanism, disulfiram has proven to be an effective clinical treatment for cocaine use disorder. In a placebo-controlled, double-blinded, randomized trial conducted by Carroll et al., cocaine-addicted patients treated with a 250 mg daily dose of disulfiram reduced their cocaine use significantly compared to patients treated with placebo [72]. Notably, disulfiram was less effective at controlling cocaine use in patients with comorbid AUD [72]. This finding was hypothesized to be due to better compliance in non-AUD participants, although specific compliance differences between alcohol users and non-alcohol users were not reported [72].

Disulfiram’s utility in cocaine addiction has also been shown to extend to patients with other comorbid substance use disorders [70]. In a small study of patients on buprenorphine maintenance treatment for opioid use disorder, disulfiram again was found to increase abstinence from cocaine use compared to placebo [70]. Notably, there was no significant difference between groups in terms of opioid abstinence [70]. Likewise, a clinical trial conducted by Kampangkaew et al. showed that cocaine- and opioid-dependent patients receiving methadone maintenance treatment experienced a significant decrease in cocaine-positive urine samples when treated with 250 mg disulfiram daily compared to placebo [73]. Interestingly, the authors also subdivided participants in each treatment group based on the concentration of dopamine transporters (DAT) in the brain as determined by genetic testing. They noted that patients with genetically higher DAT levels exhibited better treatment outcomes with disulfiram than those with lower DAT levels, indicating that patient genetics may influence treatment efficacy [73].

#### 2.3.3. Disulfiram for Crack Cocaine Addiction

Baldacara et al. assessed the effectiveness of disulfiram as an add-on therapy to motivational interviewing and group therapy for patients with crack cocaine dependence [74]. The double-blind pilot study compared treatment with 250 mg of disulfiram daily vs. placebo for 60 days [74]. At the end of the study, 87% of subjects in the disulfiram group were drug-free compared to 47% in the control group (*p* = 0.020). Disulfiram reduced the crack cocaine use dose and frequency by more than 50% [74].

### 2.4. Use in Infection

Since the 1940s, disulfiram has been investigated as a potential antiparasitic, antibiotic, and antiviral medication for infections ranging from multi-drug-resistant tuberculosis to the novel coronavirus SARS-CoV-2 [14]. Disulfiram has only limited activity against most common bacterial pathogens and may not be a realistic candidate for drug development. It is, however, active against a few pathogens including *Borrelia burgdorferi*. Investigating the use of medications other than standard antibiotics for the treatment of bacterial infections is important for two major reasons. First, the use of repurposed medications may minimize the risk of antibiotic resistance via mechanisms including virulence inhibition and immunomodulation [75], as well as increased specificity of the antimicrobial action. Secondly, utilizing an existing medication is far cheaper than developing a novel antimicrobial drug [75], costing roughly 10× less than the two to three billion dollars commonly required to develop a single new drug [76].

#### 2.4.1. Disulfiram as an Antibiotic

Disulfiram’s electrophilic nature and ability to form disulfide bonds with thiol-bearing compounds enables it to bind and modify thiophilic bacterial enzymes and cofactors. These modifications can inhibit bacterial enzymes, thereby altering the normal bacterial cell function. Known antibiotics such as fosfomycin and bacitracin utilize a similar mechanism by covalently modifying bacterial uridine diphosphate (UDP) transferases to inhibit cell wall synthesis. Based on this principle, disulfiram has been investigated in vitro for its activity against a broad spectrum of bacteria. 

Studies investigating the minimum inhibitory concentration (MIC) of disulfiram and its metabolites against Gram-positive organisms have determined that disulfiram inhibits the growth of methicillin-sensitive *Staphylococcus aureus* (MIC 8–16 μg/mL), *Staphylococcus epidermidis* (MIC 1–32 μg/mL), group A *Streptococcus* (MIC 16 μg/mL), *Enterococcus faecium* (MIC 16 μg/mL), and *Bacillus cereus* (MIC 4 μg/mL), although it is consistently less efficacious than vancomycin against these bacteria (MIC ≤ 0.5–2 μg/mL) [9,11,77]. However, for some resistant Gram-positive organisms including vancomycin-resistant *Staphylococcus aureus* (VRSA) and vancomycin-resistant *Enterococcus faecium* (VRE), disulfiram showed superior inhibition of bacterial growth in vitro compared to vancomycin [9,77].

One study noted that combination therapy with vancomycin and disulfiram exhibited synergy against vancomycin-resistant species, lowering the MIC of vancomycin alone from 128 μg/mL to 4–16 μg/mL when combined with disulfiram [77]. Another in vitro study of disulfiram + vancomycin for the treatment of VRE found the combination to be significantly more potent (MIC 0.5–4 µg/mL) than either disulfiram (16–64 µg/mL) or vancomycin (128–1024 µg/mL) alone, with synergy against all strains [78]. These studies show disulfiram’s potential as an antibiotic adjuvant for chronic or recurrent infections with vancomycin-resistant organisms. Similarly, disulfiram and its S-octyl derivative have been shown to sensitize *Staphylococcus aureus* to the effects of fosfomycin [79].

Disulfiram does not inhibit growth of most Gram-negative organisms, including *Klebsiella pneumoniae, Pseudomonas aeruginosa, Escherichia coli, Salmonella typhi,* or *Vibrio cholerae* (MIC > 32 μg/mL) [9,11,77]. This finding is believed to be due to the antagonistic effects of glutathione, which is found in abundance within Gram-negative organisms [77]. Glutathione contains a cysteine residue that reacts readily with disulfiram, cleaving it into diethyldithiocarbamate (DDTC) [77]. Unlike disulfiram, DDTC does not have a disulfide bond that can participate in the thioldisulfide exchange necessary to inhibit essential bacterial enzymes [11]. As a result, DDTC does not have antibacterial activity against even most Gram-positive organisms (MIC 16–64 μg/mL) [9,11,77]. Disulfiram has been shown to kill some Gram-negative organisms in vitro, including *Borrelia burgdorferi* (0.19–1.48 μg/mL), *Francisella tularensis* (0.50–9.50 μg/mL), and *Bartonella henselae* (2.5 μg/mL) [75]. Additionally, disulfiram has shown activity against the atypical bacteria *Mycoplasma* spp. (0.19 μg/mL). However, as the disulfiram plasma concentrations typically stabilize at 0.1–0.2 μg/mL after one week of 500 mg daily treatment, only *Mycoplasma* spp and *Borrelia burgdorferi* from this list represent possible in vivo treatment candidates [75].

Disulfiram appears to have synergistic effects with antibiotics against Gram-negative organisms. Disulfiram enhances polymyxin B’s activity against *Klebsiella pneumoniae* both in vitro and in vivo [80]. It also demonstrates synergy with meropenem against MBL-expressing carbapenem-resistant *Acinetobacter baumannii* infections, lowering meropenem’s MIC by 4- to 32-fold [81]. One explanation for disulfiram’s efficacy against drug-resistant, Gram-negative species may be its activity against New Delhi metallo-beta-lactamase 1 (NDM-1), an enzyme that confers beta-lactam resistance to many such species. Disulfiram inactivates NDM-1 via covalent disulfide bonding to NDM-1’s Cys208 residue and via its in vivo copper-bound metabolite, Cu(DTC)_2_, which oxidizes the Zn(ii) thiolate side of NDM-1 [82].

Studies have also tested the efficacy of disulfiram against sensitive, multi-drug resistant (MDR), and extensively drug-resistant (XDR) strains of *Mycobacterium tuberculosis* (TB). In vitro studies have revealed that both disulfiram and DDTC exhibit potent inhibition of both drug-sensitive and drug-resistant TB, with MICs of 0.78–1.56 μg/mL and 1.56–6.25 μg/mL, respectively [14,83]. In a mouse model of chronic TB, mice treated with 40 mg/kg of disulfiram daily for 28 days experienced significant reductions in the amounts of bacteria found in the spleen and lungs compared to controls [14]. Although the mechanism of disulfiram’s antitubercular activity remains unknown, it may be due in part to the inhibition of tubercular beta-carbonic anhydrase [14,84]. Beta-carbonic anhydrase utilizes zinc as a co-factor and is responsible for catalyzing the breakdown of carbon dioxide to bicarbonate and hydrogen ions [84]. Although studies have shown that the chelation of the zinc ion cofactor by DDTC inhibits proper enzymatic function, it has not been clearly established that this inhibition causes bacterial cell death [14,84]. The authors of a 2022 review on disulfiram’s antibiotic efficacy argue that disulfiram should still not be considered as a monotherapy for TB or non-tuberculous mycobacteria; if anything, it would be utilized alongside other agents as an “antimicrobial enhancer” in order to achieve synergistic effects [75].

When tested against zoonotic agents, disulfiram was found to be a potent inhibitor of *Bacillus anthracis* (MIC ≤ 0.5–2 μg/mL) [9,11]. Multiple mechanisms are believed to be responsible for this finding, including the thiol–disulfide exchange reaction permitting the covalent modification of bacterial enzymes, as well as metal ion chelation [11]. *B. anthracis* is known to utilize iron, copper, and zinc for its biosynthetic processes and for protection against reactive oxygen species [11]. Disulfiram could, therefore, deplete metal ions from the growth medium to levels insufficient for bacterial growth [9,11,77].

#### 2.4.2. Disulfiram in Lyme Disease

Most recently, disulfiram has received attention as a potential treatment for Lyme disease [10,85]. The spirochete *Borrelia burgdorferi sensu stricto* (*Bbss*), the primary causative agent of Lyme disease in North America, is generally highly susceptible to treatment with standard antibiotics such as doxycycline, amoxicillin, and ceftriaxone. However, 10–20% of patients who receive standard antibiotic therapy develop persistent symptoms of muscle and joint pain, cognitive slowing, and fatigue, which together comprise a syndrome known as post-treatment Lyme disease syndrome (PTLDS) [86]. The mechanisms proposed for persistent symptoms include immune dysregulation (e.g., inflammation, autoimmunity), persistent infection, abnormal neural network activation, and dysbiosis. When animal models reveal persistent *Bbss* despite antibiotic treatment, these organisms have been described as attenuated or as viable but not cultivable [87,88,89]. Most studies of *Borrelia* antimicrobial tolerance have been conducted in the United States using the *Bbss* species; the authors of this review are not aware of in vitro or animal studies confirming persistence of other species of *Borrelia*. The frequency and extent to which persister *Borrelia* or *Borrelia* remnants (e.g., peptidoglycan) are triggering immune responses that contribute to post-treatment Lyme disease symptoms is uncertain, representing an area requiring further investigation [90,91]. 

Like many other bacteria, *Bbss* is most susceptible to antibiotic treatment during its logarithmic growth phase [92]. In vitro studies have identified that after three to four days of logarithmic growth, the spirochetes enter a stationary phase characterized by a deceleration of bacterial growth and an increase in bacterial persistence despite antibiotic treatment [92]. Because of the hypothesis that the persister *Borrelia* may be responsible for the ongoing symptoms in PTLDS, finding treatments capable of eliminating persisters has become a focus of Lyme disease research [92,93].

Recent in vitro studies suggest that disulfiram is particularly effective against *Bbss* persisters. Following a screening of over 4000 FDA-approved compounds, disulfiram emerged as the most effective compound against stationary-phase *Bbss*, with 99.8% inhibition of metabolic activity at a dose equivalent to 0.38 μg/mL [94]. The mechanism of action against *Bbss* is unknown but is likely to involve the covalent modification of sulfhydryl residues on essential bacterial proteins. A mouse study found that the treatment of *Bbss*-infected mice with 75 mg/kg disulfiram daily for five days led to the reduction or clearance of spirochetes from most tissues and significantly reduced leukocyte infiltration in cardiac tissue [95]. These findings indicate that disulfiram may function both by decreasing *Bbss* colonization in host tissues and by decreasing inflammation via the manipulation of the adaptive immune response [95]. While these results appear promising, it is worth noting that a dosage of 75 mg/kg would be dangerously toxic in humans. In addition, not all studies support the conclusion that disulfiram is effective in vitro against *Bbss* persisters. A recent in vitro study showed that disulfiram was less effective than other antibiotics, such as clarithromycin and nitroxoline, when tested in a stationary-phase *Bbss* culture known to be enriched with the persister *Bbss* [96].

In 2019, the interest in the use of disulfiram in humans with persistent Lyme-disease-related symptoms despite antibiotic treatment grew when a report was published suggesting that disulfiram treatment led to the sustained resolution of symptoms in two of three individuals [85]. In 2020, a retrospective analysis of 74 patients from the same clinical outpatient practice was published describing their experience treating patients with disulfiram for Lyme-related symptoms [97]. The clinicians used an “open-ended” disulfiram dosage and duration protocol with close monitoring of patients. A retrospective analysis of the clinical series grouped patients based on their maximum dose of disulfiram and body weight; 34 were categorized in the analysis phase as having received “high-dose” treatment (≥4 mg/kg/day) and 33 as “low-dose” (<4 mg/kg/day). Of the 67 patients, 12 (17.9%) achieved “enduring remission” of greater than six months. All 12 with enduring remission had completed one or two courses of disulfiram at the higher dose, while none in the low-dose group achieved enduring remission. Furthermore, 62 of the 67 patients (92.5%) reported a meaningful reduction in some symptoms. Compared to the low-dose group, the high-dose group experienced more adverse reactions of fatigue, psychiatric symptoms, peripheral neuropathy, and elevated liver enzymes; the authors report that these symptoms resolved after the discontinuation of disulfiram, although the resolution of the neuropathic symptoms took weeks to months and one patient had mild residuum. Given the risks associated with disulfiram, the authors recommended the close monitoring of patients during disulfiram treatment. This report, while informative and carefully presented, is also limited by several factors inherent in a clinical series, including the lack of standardized outcome measures to assess the safety and treatment response, the absence of randomization and a placebo control group, and the potential bias associated with clinically treated patients wishing to please their clinician by reporting a favorable outcome. The authors highlighted several of these limitations and emphasized the need for a double-blind, placebo-controlled clinical trial to determine efficacy.

#### 2.4.3. Disulfiram as an Antiparasitic

Prior to its discovery as a deterrent in alcohol use disorder, disulfiram was originally noted for its antiparasitic properties. In the 1940s, British and Swedish researchers identified disulfiram’s ability to kill scabies and intestinal worms, both common causes of domestic animal infections at the time [1,2]. Since then, disulfiram has been a drug of interest in the treatment of parasitic infections.

Disulfiram has shown potential as a possible treatment for malaria. Scheibel et al. found that disulfiram and DDTC halted the multiplication of *Plasmodium falciparum* by inhibiting oxidase enzymes necessary for metabolism via metal ion chelation in vitro [15]. Another study found that the antimalarial activity was potentiated by the addition of copper, indicating that a disulfiram–copper complex may be toxic to the malarial parasites [98]. However, when researchers tested the serum drawn from healthy volunteers after four days of 500 mg daily doses of disulfiram, they did not find inhibition of parasitic growth [15].

Disulfiram also shows both in vitro and in vivo activities against *Giardia.* Researchers screened over 4000 FDA-approved compounds and identified disulfiram as the most effective against *Giardia* trophozoites [99]. Further investigation determined that disulfiram potently inhibits carbamate kinase, an enzyme essential for *Giardia* ATP synthesis, via the covalent modification of the enzyme’s cysteine group [19]. In vivo mouse experiments likewise showed the efficacy of disulfiram as being comparable to metronidazole, with the elimination of nearly all trophozoites following treatment [19].

There is some evidence to suggest that disulfiram could be effective against *Cryptosporidium parvum*. In a study of *C. parvum* infection in a severe combined immunodeficiency (SCID) mouse model, 1000 mg/kg of disulfiram daily for 2 weeks resulted in a 48% decrease in parasite burden compared to the control [100]. It is worth noting that this dosage drastically exceeds that which can be safely administered to human subjects; the authors suggest that it should be explored at lower concentrations in combination with other antimicrobial agents. Its efficacy may stem from its demonstrated irreversible inhibition of *C. parvum*’s inosine-5′-monophosphate dehydrogenase (Cp-IMPDH), an enzyme involved in diverse cellular processes including RNA and DNA synthesis [100]. As disulfiram is also shown to irreversibly inhibit human IMPDH, indicating poor selectivity for the parasitic enzyme, any future studies performed in human subjects must involve close monitoring of side effects.

In Sweden and Iceland, disulfiram is used in combination with benzyl benzoate (Tenutex^®^; 2%/22.5%, respectively) as a topical treatment for both scabies (*Sarcoptes scabiei*) and pediculosis (*Pediculus humanus capitis*). The treatment is highly effective in eliminating organisms after just 6–24 h of topical exposure [101]. Tenutex is also effective against permethrin-resistant *P. humanis capitis* adults and eggs [101]. Pediculosis is increasingly resistant to treatments including topical permethrin, malathion, and lindane; for example, permethrin is only 30–40% effective at treating pediculosis in some parts of the UK [101]. Treatments capable of killing resistant organisms, therefore, merit further exploration.

There is some in vitro evidence to suggest that disulfiram could be effective against the trematode genus *Schistosoma*. An early in vitro study found that disulfiram demonstrated antischistosomal activity at concentrations of 25–50 µM [102]. A subsequent study found that disulfiram and three of its metabolites (dithiocarbamic acid, dithiocarbamate *S,S*-dioxide, and thiocarbamate *S*-oxide) demonstrated antischistosomal activity, with disulfiram being more effective than its metabolites. Nonetheless, this same study identified several novel compounds based on a dithiocarbamate core that reduced *Schistosoma* motility at just 10 µM, suggesting that these novel compounds may represent more promising treatment options than disulfiram itself.

In a mouse model of *Entamoeba histolytica* amebic colitis, the combination of disulfiram and zinc (Zinc-ditiocarb complex, ZnDTC) was effective at clearing parasites and also reduced the inflammatory response and tissue damage traditionally associated with infection [103]. Importantly, this effect was shown at concentrations below those achieved by disulfiram therapy at the currently recommended doses [76]. ZnDTC was found to inhibit the COP9 signalosome, a vital multi-subunit complex in *E. histolytica*’s ubiquitin–proteasome pathway, via the inhibition of the metalloprotease activity of COP9 subunit 5 (CSN5). As CSN5 is also expressed in other organisms including *Leishmania*, *Trypanosoma*, *Toxoplasma*, and *Naegleria*, ZnDTC might demonstrate efficacy against these other pathogens via the same mechanism [76]. Disulfiram may also inhibit *E. histolytica*’s glycolytic pathways [76].

Investigators searching for trypanocidal compounds found that disulfiram caused physiologically significant reductions in the motility and infectivity of *Trypanosoma brucei*, the organism responsible for causing African trypanosomiasis. It did so by blocking the cleavage of threonine, an amino acid essential for *Trypanosoma* nutrition, and by chelating metal ions essential to the function of other parasitic enzymes in vitro [104,105]. A recent study of disulfiram and its metabolite DETC in the treatment of *Trypanosoma cruzi* (responsible for Chagas disease) found that DETC inhibited the proliferation of epimastigote forms with an IC_50_ of 1.48 µM. Administering 10 mg/kg/day each of DETC and benznidazole increased the selectivity 13-fold compared to benznidazole alone. This combination also dramatically increased the survival in infected mice at 60 days (60% vs. 10%; *p* < 0.01) and was associated with no deposition of amastigote nests in myocardium [106]. As 10 mg/kg/day is higher than could be compatible with the maximum dose of 500 mg/day in adult humans, further in vivo combination treatment studies with lower disulfiram doses (e.g., 5 mg/kg/day) may be warranted.

Disulfiram also has activity against other organisms within the Trypanosomatida order, namely within the *Leishmania* genus. It is active against amastigotes of *Leishmania donovoni,* as well as amastigotes and promastigotes of *Leishmania major* at nanomolar concentrations in vitro [22,107,108]. Peniche et al. evaluated disulfiram in an ex vivo study of mice and hamster spleens and lymph nodes following inoculation with *L. donovoni* and *L. major* [22]. The results showed that disulfiram killed intracellular amastigotes at concentrations lower than standard antileishmanial treatments such as miltefosine and amphotericin B [22]. As with *P. falciparum*, the co-administration of disulfiram with a metal ion potentiated the killing response. The addition of a divalent zinc salt improved the activity by 3- to 12-fold against *L. donovoni* and *L. major* [22]. Disulfiram’s activity against *Leishmania* is thought to stem from the disruption of the mitochondrial proton transport system used in ATP synthesis, thereby generating reactive oxygen species [22]. Given the potentiation of anti-Leishmanial activity with the addition of zinc, the creation of a toxic disulfiram–zinc complex should also be considered [22,98].

While many of the conditions discussed in this review primarily affect adults, parasitic illnesses frequently affect young children and can have devastating impacts on growth and development [109]. As most studies of disulfiram’s pharmacodynamics and pharmacokinetics have been performed in adults, these phenomena are poorly understood in children [76]. More research on the dosing and safety is likely needed before disulfiram can be administered safely to children for the treatment of parasitic diseases.

#### 2.4.4. Disulfiram as an Antiviral

Zinc plays a critical role in enzyme-catalyzed reactions in a multitude of viruses, including HIV-1, hepatitis C virus, and arenavirus [110,111]. As a result, researchers have proposed that drugs capable of removing Zn^2+^ from these necessary viral enzymes could inhibit viral replication and be potential candidates for antiviral therapy [111].

Prior to the discovery of antiretroviral therapy, disulfiram and its metabolite DDTC had been considered agents of interest in the treatment of HIV [112]. Clinical trials in HIV-positive persons found a subjectively improved clinical status among patients taking DDTC vs. placebo; however, they did not show significant changes in reverse transcriptase activity and showed mixed results for DDTC’s ability to increase CD4+ cell counts [112,113]. More recently, the finding that disulfiram reactivates latently infected CD4+ T cells in vitro has stimulated renewed interest in its use for HIV treatment [114]. These latently infected CD4+ cells act as a viral reservoir that remains inaccessible to standard therapies [115]. Reactivating these cells may provide an opportunity for the complete eradication of the virus, as these re-activated cells could then be targeted by the existent antiretroviral therapies. The proposed mechanism involves disulfiram’s inhibition of phosphatase and tensin homolog (PTEN) [115,116]. As PTEN inhibits a signaling pathway that leads to the downstream activation of HIV transcription [115,116], disulfiram’s inhibition of PTEN would, therefore, activate viral HIV transcription and enable the reactivation of latent cells [115]. In spite of this theoretical basis, a recent in vitro study examining the combination of disulfiram and maraviroc against HIV found that the addition of disulfiram conferred no additional benefit in reactivating HIV [117]. Additionally, the authors found that disulfiram was highly toxic to CD8+ T cells from patients on antiretroviral therapy, which poses limitations for future trials [117]. Additionally, none of the clinical trials to date have reported a decrease in latently infected cells in HIV patients [115]. Further clinical research is needed to investigate disulfiram’s utility in this context.

An in vitro study measuring the effect of disulfiram on viral hepatitis C replication in hepatic cells identified concentration-dependent inhibition of viral RNA replication [111]. The same study compared disulfiram in vitro to standard hepatitis C virus (HCV) medications including ribavirin and interferon-α [111]. The results showed that disulfiram and ribavirin had comparable results for the inhibition of HCV viral replication, but both performed better when combined with interferon-α [111]. Disulfiram’s activity against HCV may stem from its effects on non-structural protein 5A (NS5A), which plays an important role in HCV replication and assembly. Disulfiram removes zinc from the zinc finger site of NS5A, thereby destabilizing its structure and inhibiting its normal function [118]. While disulfiram would certainly represent a more cost-effective option than simeprevir, sofosbuvir, and ledipasvir, additional studies are needed to assess its safety and efficacy in clinical application.

Most recently, speculation emerged that disulfiram may be repurposed to reduce inflammation in individuals with severe disease from SARS-CoV-2 infection. This hypothesis stemmed from a study demonstrating that disulfiram reduced inflammation caused by sepsis in mice by blocking a protein involved in inflammation [119]. In 2021, a retrospective cohort study was undertaken to examine whether disulfiram may have had a protective effect against SARS-CoV-2 infection. The analysis included 944,127 veterans tested for SARS-CoV-2, of whom 2233 had been given disulfiram for alcoholism. After a multi-variable Cox regression to adjust for AUD diagnosis and demographics, those taking disulfiram were found to have a 34% lower incidence rate of SARS-CoV-2 infection than those not taking disulfiram. In addition, disulfiram was associated with a decreased risk of death among those infected with SARS-CoV-2 (0% of those on disulfiram vs. 3% of those not on disulfiram). While this epidemiologic study can only indicate association and not causality, the study does suggest that disulfiram could contribute to reduced incidence of SARS-CoV-2 infection [120]. One phase II clinical trial (NCT04485130) was begun in May 2021 but was suspended in May 2022 due to lower case numbers and the availability of other treatment options.

Many viral proteases, including papain-like protease (PL^pro^) and 3C-like protease (CL^pro^), are necessary for viral replication and are common among strains of coronavirus including MERS-CoV, SARS-CoV-1, and SARS-CoV-2 [20,121]. These proteases and many others contain catalytic cysteine residues that are crucial for enzymatic function [121,122]. Because disulfiram is known to bind and covalently modify these residues, researchers have investigated its ability to inhibit coronavirus activity [20,121,123,124,125,126,127]. In 2018, one in vitro study found that disulfiram inhibited proteases in the MERS-CoV and SARS-CoV-1 coronavirus strains [20]. Multiple studies have shown that disulfiram binds cysteine residues in SARS-CoV-2’s main protease (M^pro^) [123,124], although there is evidence that disulfiram acts as a “promiscuous” cysteine inhibitor that is nonspecific to M^pro^ [124]. The evidence is mixed as to whether disulfiram inhibits papain-like protease (PL^pro^), an essential enzyme involved in the generation of the replicase complex. One 2020 study found that disulfiram inhibits SARS-CoV-2 by binding to cysteine in PL^pro^ [126], whereas a 2022 study found that disulfiram had no inhibitory activity against PL^pro^ [127].

Disulfiram is known as a “zinc-ejector” and has been shown to specifically target conserved zinc-bound cysteine residues [125,126]. This mechanism allows it to target the highly-conserved non-structural proteins nsp13 and nsp14 [128], critical enzymes that function in viral replication as a helicase [129] and an exoribonuclease, respectively. A study of disulfiram against viral pseudo-particles (Vpps) of multiple SARS-CoV-2 strains showed that disulfiram potently blocked viral entry via the inhibition of the interaction between the spike protein and its ACE2 receptor [130]. These findings suggest that disulfiram could potentially demonstrate efficacy against multiple strains of SARS-CoV-2. In light of the initial results from disulfiram studies, investigators examined the activity of molecules containing thiuram disulfide and dithiobis-(thioformate) against three vital proteases involved in SARS-CoV-2 replication. They discovered three molecules (RI175, RI173, RI172) that inhibited the key proteases much more potently than disulfiram (5×, 19×, and 11×, respectively) [131]. While disulfiram has the advantage of an abundance of existing research studies and safety data, these three novel compounds were better able to specifically target key enzymes in SARS-CoV-2.

### 2.5. Use for Inflammation

Disulfiram was first studied as a potential treatment for nickel-induced hand dermatitis because of its known metal ion chelation properties [132,133]. Small double-blinded clinical trials showed the clearance of dermatitis in the majority of patients who received disulfiram compared to placebo [132,133,134]. More recently, researchers have discovered that disulfiram decreases inflammation via multiple unique mechanisms, which will be elaborated upon below.

Nuclear factor κB (NF-κB) is a transcription factor that is activated by inflammatory cytokines and reactive oxygen species and initiates the signaling pathway that responds to invading pathogens [25,135]. Studies have shown that disulfiram or its derivatives inhibit the NF-κB pathway, although the mechanism is not agreed upon [24,25]. In vitro, disulfiram’s inhibition of NF-κB appeared to be dependent on its metal ion chelation properties [25]. An in vitro experiment in bone-marrow-derived macrophages infected with murine CMV showed that disulfiram modulated NF-κB [136]. In a rat model of endometriosis, 100 mg/kg of daily disulfiram for 21 days inhibited the angiogenesis, proliferation, and expression of inflammatory cytokines via the inhibition of NF-κB and induction of oxidative stress [24]. Another in vivo study demonstrated that disulfiram was comparably effective to dexamethasone in reducing acute ocular inflammation in rats via the inhibition of NF-κB [7]. In this study, treatment with a one-time dose of 750 mg/kg of disulfiram significantly reduced the number of infiltrating cells and concentration of inflammatory cytokines in the aqueous humor [7].

One other recently identified target of disulfiram therapy is the NOD-, LRR-, and pyrin-domain-containing protein 3 (NLRP3) inflammasome [12,119,136]. Under normal physiologic conditions, the NLRP3 inflammasome initiates programmed cell death (pyroptosis) and releases inflammatory cytokines in response to pathogens [137]. The dysfunction of the NLRP3 inflammasome is implicated in a variety of inflammatory disorders including acute peritonitis, sepsis, and gout [12,119,137]. An in vitro study found that disulfiram significantly reduced NLRP3-induced pyroptosis in mouse macrophage cell lines, possibly by stabilizing the lysosome and preventing the release of cytotoxic compounds into the cytoplasm [12]. The same study also investigated disulfiram in mouse models of peritonitis and gout. Mice pre-treated with 100 mg/kg of disulfiram for three days before peritonitis induction showed significantly less inflammatory cytokine production in the serum and peritoneal fluid than control mice [12]. Mice who received disulfiram 30 min after an intra-articular injection of monosodium urate crystals showed decreased inflammatory cytokine production in the synovial fluid, decreased inflammatory cell infiltration, and decreased synovial thickening based on their histology results compared to control mice [12]. In 2020, Hu et al. published an article supporting the above findings of the disulfiram inhibition of the NLRP3 inflammasome and subsequent inhibition of pyroptosis in vitro [119]. They also evaluated disulfiram in a mouse model of lipopolysaccharide (LPS)-induced sepsis and found that at all doses of LPS induction, the disulfiram-treated mice were significantly more likely to survive or to have prolonged survival compared to controls [119].

Disulfiram also inhibits pyroptosis via its effects on gasdermin D (GSDMD), an inflammasome component that induces the formation of pores and the release of inflammatory cytokines (“cytokine storm”) [119,138]. A recent study examined the combination of disulfiram and the known anti-inflammatory molecule lactoferrin complexed into a nanoparticulate system in murine models of ulcerative colitis and LPS-induced sepsis [138]. The nanoparticles showed very high efficacy in both disease states, theorized to be due to the suppression of pyroptosis and macrophage-mediated inflammatory cytokine release [138]. Two studies examining murine models of acute pancreatitis have found that disulfiram alleviated inflammation via the inhibition of pyroptosis. One study found that this effect extended to the improvement of associated lung injury [139]. The other study specifically explored GSDMD and found that disulfiram notably decreased its expression in the endoplasmic reticulum of murine pancreatic cells [140]. In a rat model of unilateral ureteral obstruction, disulfiram pretreatment significantly inhibited pyroptosis via the inhibition of GSDMD cleavage and was able to prevent the development of renal fibrosis [141].

Disulfiram’s anti-inflammatory effects have begun to be studied in other inflammatory conditions. An in vitro study of human chondrocytes found that co-treatment with glycyrrhizic acid suppressed the chondrocytes’ inflammatory response [142]. Two disulfiram studies have been conducted in Graves’ orbitopathy (GO), a sequela of Graves’ disease involving the inflammation and swelling of orbital soft tissue as well as the fibrosis of extraocular muscles [143,144]. An in vitro model using human cells isolated from GO patients demonstrated that disulfiram inhibited hyaluronic acid production, orbital fibroblast adipogenesis, inflammation, and fibrosis [143]. In a subsequent paper, the same investigators showed that the inhibition of fibrosis and extracellular matrix (ECM) production markers was dose-dependent, with antifibrotic effects partly mediated by the extracellular-signal-regulated kinase (ERK)–snail signaling pathway [144].

### 2.6. Use in Cancer

One of the most rapidly evolving areas of research is the use of disulfiram as an anticancer agent. An October 2022 NCBI search for “disulfiram cancer” yielded 192 titles published between 2020 and 2022 compared to 110 published between 2017 and 2019. Rather than exhaustively review every type of cancer discussed, we will touch on a few major elements of disulfiram’s use in cancer treatment. Disulfiram’s potential chemotherapeutic activity was first identified in the 1970s, although it was not until recently that experimentation began to reveal the underlying mechanisms [145]. The literature now indicates that there are likely several mechanisms responsible for disulfiram’s activity against cancer cells, and these mechanisms may vary depending on the histology.

In vitro and in vivo studies have demonstrated that disulfiram’s anticancer activity is dependent on the presence of copper ions [146,147]. Both animal and human studies have shown the elevation of serum copper in individuals with cancer compared to controls [148]. As mentioned previously, disulfiram metabolite diethyldithiocarbamine (DDTC) is a potent metal ion chelator, and the chelation of metal ions leads to the formation of a DDTC–metal ion complex. It is hypothesized that elevated DDTC–copper and depleted essential copper ions are deleterious for cancer cell survival [146,147,149]. Increased levels of serum ceruloplasmin, copper’s main carrier protein, are associated with angiogenesis and have been found in various cancers [150]. Moreover, various angiogenic markers (e.g., VEGF, IL-1, IL-8) have been shown to be downregulated after the elimination of copper [150]. Recent studies have shown that combining disulfiram with copper (DSF/Cu) is effective in targeting proteasome activity [151], ALDH activity, and NF-κB activity, enhancing the cytotoxicity of various chemotherapeutic agents and overcoming resistance mechanisms [150].

Another potential mechanism for the induction of cancer cell death is the creation of oxidative stress. First, the chelation reaction between the DDTC and copper ions may release reactive oxygen species (ROS) into the intracellular environment [10,149,152]. Second, the depletion of copper and other metal ions such as zinc may inhibit important antioxidant enzymes such as superoxide dismutase, further disrupting the balance of cellular pro- and antioxidants [149]. The creation of oxidative stress may lead to damage or the death of the affected cancer cell [24]. The generation of ROS by disulfiram has been shown to activate the ROS-JNK proapoptotic pathway [151] and to induce the unfolded protein response (UPR), which triggers autophagy-dependent cancer cell apoptosis [153].

Disulfiram likely also inhibits important intracellular processes such as the ubiquitin–proteasome pathway, which is responsible for tagging and degrading misfolded proteins [145,154]. The inhibition of this system would allow for the accumulation of unnecessary or cytotoxic proteins, which could ultimately stress and kill the cell [145,154]. Large-scale screenings have identified disulfiram as a potential proteasome inhibitor, and in vitro studies have confirmed that DDTC–copper complexes prevent functional protein degradation in cancer cells [145,146,151]. The resulting aggregation of misfolded proteins has been shown in vitro to trigger a cellular heat shock response that leads to the apoptosis of the affected cell [147].

Although additional research has identified mechanisms that may be specific to particular cancer etiologies, the epidemiological data suggest that disulfiram could be a promising treatment for all cancer types [147]. A retrospective observational study of over 240,000 cases from 2000–2013 found that the cancer-specific mortality was significantly reduced for continuing users of disulfiram compared to patients who previously used disulfiram (*p* < 0.001) for all cancers, including in patients with metastatic disease (*p* < 0.001) [147]. These epidemiological data, in combination with the emerging in vitro evidence of disulfiram’s efficacy, has led to an explosion of clinical trials evaluating disulfiram in cancer patients.

The first clinical trial investigating disulfiram for cancer treatment was published in 1993 [155]. The trial compared the treatment of non-metastatic breast cancer with standard fluorouracil, adriamycin, and cytoxan (FAC) chemotherapy vs. FAC plus sodium dithiocarbamate or DDTC. The DDTC-treated group showed improved overall and disease-free survival at six years post-treatment compared to the FAC-alone group [155]. In the past decade, there have been a few additional clinical trials demonstrating modest effectiveness in certain conditions. A phase IIb trial of stage IIIb-IV non-small cell lung cancer found that the addition of disulfiram to cisplatin and vinorelbine chemotherapy led to improved survival compared to standard chemotherapy alone (10.0 vs. 7.1 months) [156]. In a phase II trial of temozolomide + DSF/Cu for recurrent temozolomide-resistant glioblastoma, 14% of patients experienced a clinical benefit, with stable disease for over six months; this finding suggests that in a small subset of patients with glioblastoma, DSF/Cu may exhibit a modest positive effect [157]. A phase II trial of patients with multiple relapsed or refractory testicular germ cell tumor (GCT) found that no patients exhibited an objective response, leading to the study’s termination in its first stage [158].

A phase I trial of DSF/Cu in patients with advanced solid liver tumors found that the regimen was well-tolerated but did not yield an objective response, with only some patients exhibiting temporary disease stabilization [159]. A phase Ib clinical trial of DSF/Cu in patients with metastatic castration-resistant prostate cancer (mCRPC) found no radiographic responses or decreases in PSA, indicating the ineffectiveness of the treatment in this population [160]. An unpublished clinical trial (NCT01118741) of patients with recurrent prostate cancer and rising PSA found that a 28-day regimen of disulfiram at 250 mg or 500 mg daily yielded a demethylation response in only 22%/30% of patients, respectively.

There are currently 22 phase I-III clinical trials listed on clinicaltrials.gov investigating disulfiram for the treatment of cancer, of which 7 are active. The diagnoses under investigation include breast cancer, prostate cancer, pancreatic cancer, liver cancer, non-small cell lung cancer, testicular germ cell tumors, multiple myeloma, sarcoma, melanoma, and glioblastoma. Given the in vitro data showing the dependence of disulfiram’s efficacy in the presence of copper ions, many of these trials are investigating the use of disulfiram plus copper alone or in addition to standard chemotherapeutic regimens in refractory cancer. While the clinical trial findings have thus far been modest at best, there is reason to hope that disulfiram could demonstrate clinical utility in combination with copper or as a supplement to standard regimens.

### 2.7. Use in Neurology

Disulfiram’s high lipid solubility enables it to cross the blood–brain barrier, indicating possible utility in the treatment of neurologic illness [29,30]. Most notably, disulfiram has been a potential drug of interest as a treatment for Alzheimer’s disease (AD) [161]. Alzheimer’s dementia is believed to result partly from the overproduction and deposition of the amyloid-β (Aβ) protein in the cerebral tissue [161]. A study published in 2018 screened over 600 FDA-approved drugs and identified disulfiram as the most promising candidate for reducing the production of the Aβ protein [161]. The subsequent in vivo study found no significant decrease in Aβ plaques within the brain tissue of mice treated with 26 mg/kg daily for two days, but did find a significant improvement in memory-related behavioral tasks in the disulfiram-treated AD model mice compared to placebo-treated mice [161].

Most current AD medications increase the synaptic concentrations of acetylcholine, a neurotransmitter known to be important for acquiring, encoding, and retrieving memories [162,163]. Additionally, patients with AD have been found to experience specific degeneration of cholinergic neurons in the brain [162,163]. A study observing the effects of chronic disulfiram administration in rats found that treatment led to increased acetylcholine concentrations and the upregulation of muscarinic receptors in the hippocampus at all tested doses [163]. The mechanism and future applications of these findings will require further investigation.

### 2.8. Adverse Reactions

Treatment with disulfiram is associated with an array of side effects ranging from minor to life-threatening [8,66,164]. This section will break down the most common and most severe reported side effects related to disulfiram use.

The most commonly reported side effects include headache, fatigue, drowsiness, papular acne, impotence, and a metallic or garlic-like taste [3]. These side effects are typically mild and reversible, rarely requiring additional treatment. As mentioned above, disulfiram causes an adverse reaction in the presence of ethanol due to its inhibition of acetaldehyde dehydrogenase, leading to the accumulation of acetaldehyde in the bloodstream [3]. The disulfiram–ethanol reaction (DER) most often occurs during the first 12 h after administration and typically consists of facial flushing, perspiration, tachycardia, nausea, and vomiting. Severe reactions can lead to hypotension, respiratory depression, cardiovascular collapse, arrhythmias, seizure, or death [3,10,165,166]. A case report of two patients who developed shock from the disulfiram–ethanol reaction found that both developed watershed infarcts in the brain and one developed probable non-occlusive mesenteric ischemia [167].

The severity of the DER is dependent on the disulfiram dose and quantity of ethanol intake, but blood alcohol levels as low as 10 mg/100 mL (equivalent to a blood alcohol content of 0.01) may cause reactions in patients on therapeutic doses of disulfiram [3]. As a result, patients are advised to abstain from the intake of any alcoholic beverages and the use of any alcohol-containing products during treatment [26]. The ubiquity of alcohol in personal hygiene and cooking products may make mild reactions common for disulfiram users. Alcohol-based hand sanitizers in particular have been implicated in such reactions [168,169,170]. A study of Japanese patients taking disulfiram found that among those who used alcohol-based hand sanitizer, 19% experienced DER and 7% experienced severe systemic reactions [168]. As the ethanol in hand sanitizers is primarily absorbed through inhalation rather than through skin contact, proper ventilation during administration may help disperse the vapor and prevent DER [169].

Elevated doses and prolonged treatment with disulfiram increase the risk of hepatotoxicity [3,171]. This toxicity ranges from a common asymptomatic rise in serum transaminases to rare fulminant hepatic failure [172]. Originally, these abnormalities were attributed to pre-existing liver conditions or alcohol intake in patients with AUD; however, subsequent studies found elevated liver enzymes after disulfiram administration in about 25% of patients without AUD [132,172,173]. Additionally, these studies identified that the hepatotoxic effects were typically reversible upon the cessation of the disulfiram treatment and then inducible upon the reintroduction of disulfiram after resolution [171,172]. The mechanism of hepatotoxicity is likely due to the production of hepatotoxic metabolites including carbon disulfide during hepatic disulfiram metabolism [171,174]. These sulfur-containing compounds can cause hepatocellular degeneration and focal or extensive necrosis [171]. As a result, the regular monitoring of liver enzymes is recommended for patients on disulfiram, regardless of the indication [3].

Disulfiram-induced peripheral neuropathy has been described in case reports dating back to the 1950s [175]. The incidence of this side effect is thought to be rare based on a national study analyzing reports to the Danish Committee on Adverse Drug Reactions [176]. The typical symptoms include distal paresthesias, numbness, weakness, and decreased pain and temperature sensations [175,177,178]. Nerve conduction studies have identified disulfiram neuropathy as a distal axonal neuropathy, with the neurological examination indicating the involvement of both large and small nerve fibers [175,177,179]. The development of neuropathy appears to be dose-dependent and is most commonly reported in patients taking the maximum dose of 500 mg/day, although some cases report neuropathic symptoms at doses as low as 250 mg/day [175,177,178,179]. The exposure duration for these cases ranges from 10 days to 30 years, with a mean of 5.5 months [179]. Similar to hepatic toxicity, the mechanism of neuronal toxicity is attributed to the accumulation of toxic metabolites such as carbon disulfide in nerves, causing axonal damage [177,179]. The published cases report the complete or nearly complete reversibility of neuropathy symptoms upon the discontinuation of disulfiram [175,177,178,179].

Disulfiram and its metabolites, which cross the blood–brain barrier, have been reported to have adverse effects on the central nervous system. One such side effect is psychosis, documented in case reports dating back to the 1960s. Disulfiram inhibits dopamine-β-hydroxylase, thereby increasing the dopamine concentration in the central nervous system [10,180]. Dopamine excess may lead to psychosis and movement disorders such as choreoathetosis. A study of psychiatric complications after disulfiram among 605 alcoholic patients randomly assigned to a 250 mg daily group, 1 mg daily group, or a no-disulfiram control group showed no significant difference in the incidence of psychiatric complications among the three groups; these results suggest that if psychiatric complications occur following disulfiram use, the incidence at a dose of 250 mg/day must be very low [180]. Cases of disulfiram-induced psychosis, however, have been reported among individuals taking disulfiram for alcohol cessation, more often in males than females [66]. Among 17 such cases described in a 2017 review, the treatment durations ranged from three days to eight months, and the daily doses ranged from 250 to 500 mg [66]; the most common symptoms were persecutory delusions, although auditory hallucinations, thought blocking, and catatonia were also reported [66]. Similar to other side effects, disulfiram-induced psychosis is reversible in almost all cases upon drug discontinuation [66,67]. Disulfiram-induced psychosis may be more common at doses of 500 mg/day than at lower doses. In a case series of 52 hospitalized individuals treated with disulfiram for alcohol abuse or dependence at 250 mg twice daily, six (11.5%) developed psychotic symptoms; these symptoms remitted after disulfiram discontinuation [181]. Cases of disulfiram-induced manic episodes have also been reported, even among individuals without a personal or family history of a psychotic or mood disorder [182]. Given disulfiram’s known toxicities, some researchers have proposed that an altered mental status may stem from toxic encephalopathy rather than drug-induced neurotransmitter alterations, although this theory has not been further elucidated [66,180,183].

Other CNS adverse effects have been reported. One rare side effect is optic neuritis [184,185,186], which is dose-related [184] and may resolve slowly over many months or be irreversible [186]. Disulfiram has also been associated with rare cases of fulminant encephalopathy and CNS abnormalities upon MRI [8,187]; both of these cases led to hospitalization and death from complications shortly thereafter. In one of these cases [187], the individual may have been taking higher than the prescribed disulfiram dose of 375 mg/day. In the second case, the individual had been given an extremely high dose of disulfiram (one gram subcutaneously) [8].

Disulfiram has additional rare side effects. One such side effect is myopathy. A study in mice found that disulfiram administration led to increased serum myoglobin and LDH, indicative of myocyte necrosis [188]. Disulfiram also caused elevations in cardiac troponin I, indicative of specific damage to the cardiac myocyte [188]. Beyond the commonly reported side effect of papular acne, other dermatologic side effects have been rarely reported. A case report described a 48-year-old patient who developed symmetrical drug-related intertriginous and flexural exanthema (SDRIFE), a rare and benign type-IV hypersensitivity reaction, after taking disulfiram at 500 mg daily for two weeks [189]. Other known dermatologic side effects include exfoliative dermatitis and pruritus, which can occur with or without a rash [190]. One extremely rare hematologic complication is methemoglobinemia, described in a case report of a 35-year-old man who had been taking disulfiram at 200 mg/day for one week. The authors stated this was only the second such case of which they were aware [191].

Lastly, disulfiram can inhibit the metabolism of other drugs in the liver via the inhibition of cytochrome P450 reductase. As a result, treatment with disulfiram, particularly if daily, may increase serum levels of drugs such as warfarin and phenytoin to toxic levels [28,192,193,194]. A study of cisplatin and disulfiram vs. cisplatin alone found that the former group exhibited greater ototoxicity (*p* < 0.005); as there is no literature demonstrating ototoxicity from disulfiram alone, this finding suggests that disulfiram may affect cisplatin metabolism and thereby magnify its ototoxicity [195]. Physicians prescribing disulfiram should evaluate it for the presence of possible toxic drug–drug interactions.

## 3. Discussion

This review provides an overview of the promising potential applications of disulfiram in clinical medicine. As an FDA-approved medication, disulfiram can be efficiently repurposed and may offer a faster solution to the above clinical problems than the creation of a novel drug. However, caution is recommended. Despite the many positive findings explored in this review, there remain barriers to immediate clinical application.

Firstly, clinical trials do not always corroborate the in vitro and in vivo findings. Many of the above studies have identified potential uses based on disulfiram’s performance in the laboratory setting, but amongst most of the categories discussed, few studies have tested its performance clinically.

Secondly, while in vivo studies use animal models that are representative of human diseases, challenges exist in applying such data. The daily doses of disulfiram used in the above mouse and rat studies ranged from 10 mg/kg to 1000 mg/kg; scaled linearly, these doses equate to 0.70 g–70 g in the average 70 kg adult and exceed the maximum allowable daily dose of 500 mg [7,161]. More accurate conversions of animal dosing to human dosing may be calculated by normalizing the dose to the body surface area [196]. Using one such conversion formula (Dose_Human_ = Dose_Animal_ ∗ [Km_Animal_/Km_Human_]) and viewing most of the studies as having been conducted in mice, the human-equivalent doses tested in the above studies ranged from 0.81 mg/kg–81 mg/kg or 0.057 g–5.7 g in the average 70 kg adult [196]. Although the doses at the lower end of this range are safe for human use, many of the doses found to be efficacious in animal models would be dangerous in clinical applications. As a result, caution must be used when interpreting in vivo animal studies.

Thirdly, disulfiram’s clinical utility is hindered by the wide range of its side effects, most notably the disulfiram–ethanol reaction. Disulfiram treatment requires complete abstinence from alcohol and the avoidance of a wide range of household products, which may prove burdensome or impossible for many patients. Disulfiram can also lead to rare but serious side effects (including hepatotoxicity, peripheral neuropathy, optic neuritis, and psychosis) and can magnify the toxicity of concomitant medications via the inhibition of cytochrome P450 reductase. Physicians prescribing disulfiram must be careful to evaluate it for the presence of drug–drug interactions.

Lastly, the high inter-subject variability in the plasma levels of disulfiram and its metabolites poses a challenge in clinical practice. Such variability could explain why some patients experience no disulfiram–ethanol reaction over a dose range of 200–300 mg/day [54], while others may experience rare and life-threatening adverse events at similar doses. This phenomenon also raises the possibility of inter-subject variability in disulfiram’s efficacy for all aforementioned uses; even if disulfiram is proven to be useful in clinical trials, the benefits may not extend to all patients. Because there have been only a few pharmacokinetic studies performed in humans, ambiguity regarding the inter-subject variability remains, thereby impeding our understanding of disulfiram’s efficacy and safety.

A narrative review was chosen for this article rather than a systematic review, given the breadth of the topic and the goal of providing a reader-friendly format to address various potential clinical applications and safety concerns related to disulfiram for human diseases. Future investigators should consider focusing on a specific question within the broad literature on disulfiram; this would enable a systematic review, which has the advantages of reducing bias by including all studies on a particular topic and critically appraising and synthesizing the results using explicit methodologic approaches.

## 4. Methods

For this narrative review, a PubMed search was conducted for articles addressing in vivo studies of disulfiram with an emphasis on drug repurposing for the treatment of human diseases. The key search terms were “disulfiram” and “Antabuse”. An additional search was conducted in clinicaltrials.gov to identify research trials in humans. Within this framework, we further conducted topic-specific searches (e.g., “disulfiram cancer”) to identify papers relevant to various applications. While we focused on human studies, we also included animal studies and in vitro studies when necessary to highlight important mechanisms and safety issues. The selection criteria were based on the authors’ evaluation of the papers’ impacts based on the study design and relevance to our study topics, with a particular focus on articles providing insight into human clinical applications.

## 5. Conclusions

In conclusion, this review has highlighted many possible clinical applications of disulfiram as well as many challenges, particularly those related to pharmacokinetic variability and safety. Further research will help to determine disulfiram’s full clinical potential.

## Figures and Tables

**Table 1:** Administration of disulfiram and the PK parameters of the metabolites.

	Drug Administered	Route	Dose	Metabolite Measured in Plasma	C_max_ (μg/mL)	t_1/2_ of the Metabolite Measured	Reference
1	Disulfiram	PO	250 mg	Disulfiram	0.3, 0.4	7 h	[36]
				DDTC	0.7, 1.4	15 h	
				DDTC-Me	0.3, 1.2	22 h	
				Diethylamine	1.7, 3.8	14 h	
				CS_2_	22, 24	9 h	
				CS_2_ (breath)	37, 44	13 h	
2	Disulfiram	PO	400 mg	DDTC-Me	NA	6 h	[50]
3	Disulfiram	PO	400 mg	DDTC-Me	0.05	6 h	[34]
				DETC-Me	0.04	11 h	

C_max_—the maximum concentration detected in the plasma; PO—oral; NA—data not available. The initial C_max_ values in # 1 represent a single dose and the later ones represent the measured quantity after repetitive doses.

## Supplementary Materials

The following supporting information can be downloaded at: https://www.mdpi.com/article/10.3390/antibiotics12030524/s1. Table S1: Human Treatment Studies and Clinical Series of Disulfiram for Various Disease Indications.
