# Supplementary material for: Disulfiram: Mechanisms, Applications, and Challenges
Source: Antibiotics (Basel). 2023 Mar 6;12(3):524. doi: 10.3390/antibiotics12030524 (PMC10044060; doi:10.3390/antibiotics12030524)
Supplement: Supplementary file 1 [file antibiotics-12-00524-s001.zip › antibiotics-2195052-supplementary.pdf]

Table S1.Human Treatment Studies and Clinical Series of Disulfiram for Various Disease Indications.

Paper	Country	Study Type	Population / Setting	N=	Intervention (Dose/Duration)	Results	Limitations
**A. Substance Use Disorders**							
Gerrein et al., 1973, Disulfiram Maintenance in Outpatient Treatment of Alcoholism [58]	USA	Clinical study with randomization	Patients newly admitted to the outpatient Alcoholism Clinic of Boston City Hospital.	121	Patients were randomly assigned to one of 4 treatment/distribution methods: 1) DSF 250mg daily given 1x/wk 2) DSF 250mg daily given 2x/wk under supervision (maintenance) 3) no DSF, attending 1x/wk 4) no DSF, attending 2x/wk. Patients who refused DSF were told to come either 5) 1x/wk for counseling or 6) 2x/wk for counseling and group discussion. All patients were offered standard counseling, psychotherapy, and medication.	40.5% of the alcoholic outpatients in the clinic agreed that if offered disulfiram, they would take it. The group receiving the daily DSF pills 2x/wk under supervision was shown to be significantly superior in keeping patients in treatment and encouraging their sobriety during the 8 week follow-up period. There was no difference in clinic attendance among the remaining five groups.	Small sample size per group, no consent about randomization.
Fuller et al., 1986, Disulfiram Treatment of Alcoholism: A Veterans Administration Cooperative Study [57]	USA	Controlled, blinded (double-blind for dose of medication condition), multicenter study	Male patients with alcoholism presenting for treatment at 9 Veterans Administration health centers.	605	Randomly assigned to 250mg DSF, 1mg DSF, or no DSF (riboflavin); bimonthly treatment assessments were done for one year. All received counseling.	There were no significant differences in total abstinence, time to first drink, social stability, or employment. Among patients who drank and had complete assessment interviews, those in the 250-mg group reported significantly fewer drinking days than those in the other two groups. Adverse reactions were uncommon. Psychiatric problems were seen in 11 patients (5 in 250mg DSF group, 3 1mg DSF, 3 riboflavin.) No patients developed severe toxic hepatitis or peripheral neuropathy. Study medication was discontinued in 4 patients because of an increase in serum ALP or AST (3 in 250mg DSF group, 1 in riboflavin group).	No correction for multiple comparisons across a number of variables
George et al., 2000, Disulfiram versus placebo for cocaine dependence in buprenorphine-	USA	Preliminary double-blind placebo-controlled clinical trial	Patients with concurrent opioid	20	Subjects were induced onto buprenorphine maintenance then randomized to DSF treatment for 12 weeks (250 mg/day; n=11 randomized of whom 8 completed) or placebo (n=9	Compared to placebo, the DSF group had significantly greater total number of weeks abstinent from cocaine and significantly lower number of days to achieving 3 weeks of continuous cocaine abstinence. DSF appeared to be well tolerated and there were no reports of DSF	Small sample size.

maintained subjects: a preliminary trial [70]			dependence and concurrent cocaine use.		randomized of whom 7 completed) treatment for 12 weeks.	reactions with alcohol use. No direct liver toxicity of DSF treatment was observed during the monthly monitoring of liver function tests.	
Carroll et al., 2004, Efficacy of disulfiram and cognitive behavior therapy in cocaine-dependent outpatients: a randomized placebo-controlled trial [72]	USA	Randomized, placebo-controlled, double-masked (for medication condition)	Individuals meeting the criteria for current cocaine dependence. Community-based outpatient substance abuse treatment program.	121	Patients received either DSF (250 mg/day) or placebo. 2x2 factorial trial with 4 treatment conditions: DSF + cognitive behavioral therapy (CBT), DSF + interpersonal psychotherapy (IPT), placebo + CBT, placebo + IPT.	Participants assigned to DSF reduced cocaine use significantly more than those assigned to placebo. Those assigned to CBT reduced cocaine use significantly more than those assigned to IPT. Benefits of both DSF and CBT were greatest for participants not alcohol dependent at baseline and those fully abstinent from alcohol during treatment. Adverse effects experienced by participants who received DSF were mild and were not considerably different from those experienced by participants who received placebo.	High attrition (50% of individuals who began treatment did not complete it).
Baker et al., 2007, Disulfiram Effects on Responses to Intravenous Cocaine Administration [71]	USA	Randomized, double-blind, placebo-controlled, within-subject study	Non-treatment-seeking, cocaine-dependent volunteers. University-affiliated medical center.	9	DSF doses included 0 mg (placebo), 62.5 or 250 mg/day on days 1–6. On days 4–6, participants received AM DSF dose 2h prior to scheduled administration of IV cocaine placebo, 0.25mg/kg (*n*=9), or 0.5mg/kg (*n*=3) over 1 min. A 7-day washout occurred between DSF conditions.	Following active DSF treatments and cocaine 0.25mg/kg administration, plasma cocaine AUC significantly increased and cocaine clearance significantly decreased. Similarly significant reduction in cocaine clearance occurred for the 0.5 mg/kg cocaine dose among those on DSF. Neither dose of DSF with cocaine altered participants' cardiovascular responses relative to cocaine alone. Following cocaine 0.25 mg/kg, 'any high', 'cocaine high', and 'rush' significantly decreased with either DSF dose. DSF decreased cocaine clearance without toxicity.	Small sample size. Lack of inclusion of a smoked cocaine group.

Baldaçara et al., 2013, Could disulfiram be a new treatment for crack cocaine dependence? A pilot study [74]	Brazil	Double-blind randomized placebo-controlled pilot study	Male subjects 18-40 years old with a diagnosis of crack cocaine dependence who sought out care at an addiction treatment center.	30	15 subjects received 250mg/day DSF for 60 days and 15 subjects (matched by gender, age, and diagnosis) received placebo. This was as add-on treatment to motivational interviewing and group therapy.	DSF significantly reduced drug use frequency compared to placebo. DSF also promoted reduction of mean drug dose used per day compared to placebo. Subjects in the DSF group were significantly more likely to be drug-free at the end of the study (87% vs. 47%). 3/15 subjects experienced side effects related to DSF, including headache, slurred speech, and drowsiness, while 1/15 (6.7%) reported headache with placebo. DSF did not cause a significant elevation of AST or ALT.	Small sample size, urine testing for cocaine conducted only 2x (requiring additional self-reports on use)
Kampangkaew et al., 2019, Pharmacogenetic Role of Dopamine Transporter (SLC6A3) Variation on Response to Disulfiram Treatment for Cocaine Addiction [73]	USA	Randomized, placebo-controlled trial	Cocaine and opioid co-dependent patients at two academic medical centers.	67	All patients were stabilized on methadone at 60 mg/day and then randomly assigned to DSF (250mg/day) or placebo for 12 weeks. Supervised urine samples were collected 3x/wk and tested for the cocaine metabolite benzoylecgonine. Both groups received weekly CBT. The *SLC6A3* (*DAT1*) 40 bp 3′ UTR VNTR variant was genotyped and its role in moderating DSF efficacy for cocaine dependence was evaluated.	In the DSF group, cocaine-positive urines dropped significantly more among the 10,10-repeat genotype group (from 78% to 48%) than among the 9-repeat carrier group (80% to 75%). In the placebo group, no difference was observed in cocaine positive urines between the 10,10-repeat genotype and the 9-allele carrier patients. On disulfiram therapy for cocaine dependence, patients with genetically higher DAT levels had better treatment outcomes than those with lower DAT levels.	Small sample size, some patients had a history of alcohol abuse (which may have reduced cocaine usage).
**B. Infectious Disease**							
Hørding et al., 1990, Lack of immunomodulating effect of disulfiram on HIV positive patients [113]	Denmark	Double-blind randomized placebo-controlled pilot study	HIV antibody (+) homosexual men with moderate	15	Participants received daily doses of 100 mg or 400 mg of DSF or placebo, for 4 weeks.	No significant effect of DSF on immunological, hematological, biochemical or clinical variables was observed in this short-term trial. Side effects were reported by 4 DSF patients; 2 had foul breath, 1 also a slight depression, 1 epigastric pain, and 1 epigastric upset and headache. 1 placebo patient had fever during Week 3 of the study, and one treated with 100 mg DSF had a transient 'flu feeling' after 2 weeks.	Small sample size, homogeneous sample

			immunodeficiency.				
Spivak et al., 2014, A pilot study assessing the safety and latency-reversing activity of disulfiram in HIV-1-infected adults on antiretroviral therapy [51]	USA	Open-label, single-arm, pilot clinical trial	HIV-1-infected individuals on stable suppressive antiretroviral therapy	16	All patients received 500 mg/day of DSF for 14 days.	DSF was safe and well tolerated; Observed adverse events were mild to moderate (grades I and II toxicity). There was high inter-subject variability in plasma DSF levels. The latent reservoir of HIV-1 did not change significantly, nor did residual viremia change significantly compared to baseline.	Lack of control group, no randomization, small sample size.
Lee et al., 2019, Population Pharmacokinetics and Pharmacodynamics of Disulfiram on Inducing Latent HIV-1 Transcription in a Phase IIb Trial [44]	USA	Phase 2 dose-escalation trial	HIV+ participants on antiretroviral therapy (ART)-suppressed	30	Participants were administered 500 mg, 1,000 mg, or 2,000 mg of DSF for 3 consecutive days (n=10 in each group). DSF and four metabolites were measured by UPLC-tandem mass spectrometry. Changes in activated viral transcription (CA-US and plasma HIV RNA) were quantified by polymerase chain reaction (PCR) and analyzed in NONMEM.	DSF was well-tolerated. DSF activated viral transcription as measured using cell-associated unspliced (CA-US) and plasma HIV RNA. A pharmaco-kinetic model demonstrated nonlinear elimination kinetics. The fitted median AUC values for 72 hours were 3,816, 8,386, and 22,331 mg*hr /L for doses of 500, 1000, and 2000 mg. Higher exposure predicted significantly greater increases in CA-US (E_{max} = 78%, AUC_{50} = 1,600μg*hr/L) but not plasma HIV RNA.	Small sample size.
Liegner, 2019, Disulfiram (Tetraethylthiuram Disulfide) in the Treatment of Lyme Disease and Babesiosis: Report of Experience in Three Cases [85]	USA	Retrospective case series	Patients treated clinically with DSF due to persistent symptoms despite prior antibiotic therapy for Lyme disease.	3	Patient 1: 90-day; DSF 500 mg/day.<br>Patient 2: 6 weeks; 500 mg/day.<br>Patient 3: 4-months; started at 125 mg every other day and gradually increased to 500mg/day for last 2 months.	Three patients were able to discontinue treatment with antimicrobial agents for periods of observation of 6–23 months following a finite course of treatment with DSF alone. DSF treatment was associated with episodes of marked fatigue in patients 2 and 3. Two patients were briefly hospitalized; one for psychiatric reasons shortly after completing the 90-day course and the other due to a syncopal episode with concussion after a 6-week DSF course (thought to be related to interaction between DSF and tricyclic antidepressant agents).	Small # of cases in this retrospective chart review, no control comparison, no standardized outcome measures.

Gao et al., 2020, "Repurposing" Disulfiram in the Treatment of Lyme Disease and Babesiosis: Retrospective Review of First 3 Years' Experience in One Medical Practice [97]	USA	Retrospective case series	Patients treated clinically with DSF due to persistent symptoms despite prior antibiotic therapy for Lyme disease.	67	67 patients received DSF, of whom 13 patients (19.4%) received more than one course of DSF. DSF dose and duration varied based on clinical history, tolerance, and preference, with some maintained at very low doses. Duration varied from 6 weeks to greater than 16 months. Retrospective analysis enabled division into two categories depending on the average daily DSF dosage: "high dose" (i.e., ≥4 mg/kg/day) and "low dose" (i.e., <4 mg/kg/day).	12/67 (17.9%) patients had "enduring remission" defined as feeling well and not receiving additional antimicrobial therapy; all 12 were in the "high dose"" category. 62/67 patients (92.5%) reported benefit which they attributed to DSF. The higher dose group experienced more adverse reactions, including fatigue (66.7%), psychiatric symptoms (48.5%), peripheral neuropathy (27.3%), and mild-moderate liver enzyme elevation (15.2%). Adverse psychiatric reactions resolved quickly when medication was discontinued, while neuropathy resolution took weeks to months with 1 patient experiencing continued mild residuum.	Retrospective chart review, no control comparator group, no standardized outcome measures.
**C. Inflammatory/Dermatologic Disease**							
Kaaber et al., 1979, Antabuse treatment of nickel dermatitis. Chelation--a new principle in the treatment of nickel dermatitis [133]	Denmark	Single-arm treatment study	Nickel-hypersensitive patients with chronic, dyshidrotic hand eczema	11	Patients whose eczema was increased by oral challenge with 0.6-2.5 mg nickel were then treated with 100 mg DSF 2-4x daily for 4-10 weeks.	9 patients experienced a dermatitis flare shortly after initiating treatment. During treatment, dermatitis cleared in 7 patients, improved in 2 patients, and was unchanged in 2 patients. 6 patients had a flare when treatment was stopped. 7 patients had side effects (e.g,. fatigue, headache, and dizziness), leading to discontinuation of DSF for 4 patients. Most side effects were mild and resolved upon reduction or discontinuation of DSF. During treatment, high nickel levels were found in urine and serum.	Small sample size, no control group, no randomization.
Kaaber et al., 1983, Treatment of nickel dermatitis with Antabuse; a double blind study [132]	Denmark	Double blind, placebo-controlled trial	Patients with hand eczema and nickel allergy	30	30 enrolled and 24 completed. DSF dose gradually increased from 50 to 200 mg daily with maximum dose given for 6 weeks. Changes observed during the study were analyzed using these parameters: scaling, frequency of flares, erythema, area involved, and number of vesicles.	During treatment, the dermatitis healed in 5/11 patients in the DSF group vs. 2/13 in the placebo group. Differences in other parameters met statistical significance only for scaling and flare frequency. The difference between groups on the sums of parameters was not statistically significant. 2 patients treated with DSF developed signs of hepatic toxicity which was biopsy confirmed in one.	Small sample size.
Ashimav Deb Sharma, 2006, Disulfiram and low nickel diet in the management of hand	India	Single-blinded treatment study	Patients with chronic vesicul	21	Patients were randomly divided into a study group (n=11) and placebo group (n=10). The study group was given a 4-week course of oral DSF (125 x 1 week and 250 x 3 weeks); they started low-	After 4 weeks, hand eczema cleared significantly more often in the DSF + LND group (10/11; 90.9%) than the placebo group (1/10; 10%). Mild relapse was noted in 5 patients during the follow-up period. DSF was well-tolerated by 6 patients (54.5%). Side effects were metallic	Not double-blinded, small sample size.

eczema: A clinical study [134]			ar hand eczema with nickel sensitivity		nickel diet (LND) 2 weeks prior to initiating DSF therapy and continued till the end of follow-up. The placebo group continued with normal diet and received 4 weeks of daily lactose tablet.	taste (n=3), mild drowsiness (n=2) and anorexia. 3 of 11 (27.3%) showed mild elevation of liver enzymes. LND and short course of oral DSF therapy was helpful for the control of chronic hand eczema in nickel-sensitive individuals.	
**D. Cancer**							
Verma et al., 1990, A randomized phase II study of cisplatin alone versus cisplatin plus disulfiram [195]	Canada	Prospective randomized study	Patients with cisplatin-sensitive malignancies	53	Patients were assigned to cisplatin (CP) 100 mg/m2 alone (group I) or CP 100 mg/m2 + oral DSF 2,000 mg/m2 (group II).	Of the 30 evaluable patients (16 Group I, 14 Group II), only one in group II achieved complete response. When response rate, median survival, and time to progression were compared between groups, no statistically significant differences were found. Patients in group II had higher grades of nausea, vomiting, and ototoxicity. Nephrotoxicity was not different between groups.	High percentage of participants (43.4% of those randomized) were inevaluable for response.
Nechushtan et al., 2015, A Phase IIb Trial Assessing the Addition of Disulfiram to Chemotherapy for the Treatment of Metastatic Non-Small Cell Lung Cancer [156]	Israel	Phase II, multicenter, randomized, double-blinded study	Newly diagnosed non-small cell lung cancer patients with either stage IV or "wet IIIb" disease	40	All patients received chemotherapy (cisplatin and vinorelbine) for six cycles. Half of patients were assigned to also receive DSF 40mg 3x/day. DSF was continued after the six cycles of chemotherapy.	Patients were treated for >2 cycles, half w/ and half w/o DSF. Significantly increased survival was noted for the experimental group (10 vs. 7.1 months), but the higher response rate (46% vs. 37%) in the DSF group was not statistically different. Quality of life did not differ between groups. Notable were 2 long-term survivors, both in the DSF group. Adding DSF to combined cisplatin/vinorelbine was well-tolerated.	Small sample size.
Huang et al., 2019, A multicenter phase II study of temozolomide plus disulfiram and copper for recurrent temozolomide-resistant glioblastoma [157]	USA	Open-label, single-arm phase II study	Patients with recurrent TMZ-resistant glioblastoma multiforme	21	Standard monthly TMZ plus concurrent DSF 80mg PO TID + Cu 1.5mg PO TID. The primary endpoint was objective response rate (ORR), and secondary endpoints included progression-free survival (PFS), overall survival (OS), clinical benefit (response or stable disease for at least 6 months), and safety.	Median duration of DSF/Cu was 1.6 cycles. The ORR was 0%, but 14% had clinical benefit (stable disease or objective response). Median PFS and OS were 1.7 months and 7.1 months respectively. One patient (4%) had dose-limiting toxicity (grade 3 ↑ALT). Adding DSF + copper to temozolomide for TMZ-resistant isocitrate dehydrogenase (IDH)-wild type GBM appears well-tolerated. However, DSF + copper has limited activity for unselected IDH-wild type glioblastoma; it also does not appear to restore TMZ sensitivity.	No blinding, no control group, small sample size.
Kelley et al., 2021, A Phase 1 dose-escalation study of disulfiram and copper gluconate in patients with advanced solid tumors involving	USA	Dose escalation study to determine maximum tolerated dose or maximum administered dose of copper gluconate with a fixed DSF dose.	Adult patients with metastatic solid	21	Four doses of copper gluconate were tested in a dose escalation design (2, 4, 6, 8 mg of elemental copper). DSF 250 mg was administered daily in 28-day cycles. Evaluation focused on dose limiting toxicities and response. Protein *S*-	Among 16 with evaluable data, DSF with a maximum of 8 mg of copper was well-tolerated with no dose limiting toxicities. Five Grade 3 toxicities were observed (↑AST, ↑ALP, fever, fatigue, anorexia). Stable disease was noted in 4 patients (longest disease control =116 days). Median duration of treatment was 55 days. Discontinuation	No blinding, no control group, small sample size.

the liver using S-glutathionylation as a biomarker [159]			tumors to the liver and an expected survival of at least 3 months.		glutathionylation was included as a pharmacodynamic marker.	reasons included disease progression, functional decline, and disease-associated death. Serum protein *S*-glutathionylation was increased with treatment. No partial or complete responses were seen.	
Mego et al., 2022, Phase II study of Disulfiram and Cisplatin in Refractory Germ Cell Tumors. The GCT-SK-006 phase II trial [158]	Slovakia	Phase II study	Patients with multiple relapsed/refractory germ cell tumors.	12	Patients received DSF 400 mg/day and cisplatin in 21 day cycles until progression or unacceptable toxicity; cisplatin was administered on day 1 and 2 of each 3 week interval at dose 50 mg/m^2 day. Investigators tracked both progression-free survival and overall survival.	0 patients attained an objective response to treatment. Additionally, cisplatin sensitivity was not restored. Study was terminated in the 1st stage. Median overall survival was 2.9 months and median PFS was 1.4 months. 2 patients (16.7%) had disease stabilization for 3+ months. . Treatment was well tolerated; however, 5 (41.7%) patients had marked fatigue, 4 (33.3%) had thrombocytopenia, 3 (25.0%) had anemia, while 2 (16.7%) experienced neutropenia, nausea, and infection.	No randomization, no control group, small sample size.
Zhang et al., 2022, Prospective clinical trial of disulfiram plus copper in men with metastatic castration-resistant prostate cancer [160]	USA	Phase 1b prospective clinical trial	Patients with metastatic castration-resistant prostate cancer	9	Patients were divided into Cohort A (nonliver/ peritoneal metastases) and B (liver and/or peritoneal metastases). All received disulfiram 80 mg 3 times daily with IV $CuCl_2$ which was given weekly for 3 doses followed by daily oral Cu gluconate until disease progression. Plasma levels of DSF and metabolite Me-DDC levels were measured. DSF and Me-DDC were then assessed for In vitro cytotoxicity to DSF and Me-DDC were then assessed in three PC cell lines.	Nine patients with mCRPC (6 in cohort A, 3 in cohort B) were treated; none had confirmed PSA declines or radiographic responses. Median OS was 8.3 months and median PFS was 2.8 months. Me-DDC was measurable in all samples , whereas DSF was not; Me-DDC was not cytotoxic in vitro. No grade 4/5 adverse events were observed. Common events were fatigue and psychomotor depression. Due to rapid metabolism of DSF into its inactive metabolite Me-DDC, the study concluded that oral DSF is not an effective treatment for mCRPC.	Small sample size.
